# Acquiring bifidobacteria species from formula-fed and breast-fed newborns: identifying, quantifying and creating an antibiogram

**DOI:** 10.1099/acmi.0.000590.v3

**Published:** 2023-08-08

**Authors:** Rajagopal Kammara, Anagha Nellikka

**Affiliations:** ^1^​ Department of Microbiology and Fermentation Technology, Council of Scientific and Industrial Research (CSIR) - Central Food Technological Research Institute (CFTRI), Mysore, Karnataka, India

**Keywords:** antibiogram, *Bifidobacterium adolescentis*, f6ppk, RFLP, xfp

## Abstract

After examining the *Bifidobacterium* spp. population in faeces samples from breast-fed and formula-fed infants, an antibiogram was created. The prevalence of *Bifidobacterium* spp. in faeces was determined using common bacterial growth media, including Man Rogos Sharpe (MRS), Brain Heart Infusion (BHI), Luria Bertani (LB) broth and Bifidobacteria agar. According to the findings, formula-fed babies had a low population of *Bifidobacterium* spp. in their stools while breast-fed babies had a high population. By using phylogenetic analysis of the 16S rRNA and *xfp* (xylose/fructose 6-phosphate phosphoketolase) genes, and RFLP mapping of *Bifidobacterium* isolates, it was possible to identify a new and unique *Bifidobacterium* species. The intensity of the reddish brown colour produced during the F6PPK (fructose 6-phosphate phosphoketolase) assay is an accurate indicator of the proportion of various bifidobacteria present. Bifidobacteria agar media produced the greatest amounts of bifidobacteria diversity and recovery. Small (SCV) and Big colony variations (BCV) were formed during growth on different media. The various antibiotic MIC values changed depending on the use of different media, growth circumstances, bile salt treatment and low pH. The findings of this study demonstrate that test conditions also impact the diversity of microbiological conditions that distinguish between resistant and susceptible bacteria.

## Data Summary

NCBI submissions of bifidobacterial isolates are FN600543, FN600542 and FN600541. These accession numbers were used to identify the nucleotide data that were provided for the GenBank database.Table S1, available in the online version of this article, contains the list of primers used in this study.

## Introduction

According to research, breast-fed children are healthy and resistant to infectious diseases because probiotic bacteria such as bifidobacteria and lactobacilli prevent the growth and amplification of disease-causing pathogenic bacteria in the intestine [1]. The primary goal of breastfeeding is to preserve and amplify the good gut microbiota. By preventing the formation and spread of potentially harmful bacteria, breastfeeding supports a healthy bowel. Very common bifidobacterial species reported in infant faecal material are *Bifidobacterium infantis, Bifidobacterium longum, Bifidobacterium bifidum, Bifidobacterium adolescentis* and *Bifidobacterium breve *. *Bifidobacterium catenulatum* is quite infrequently seen. By precisely quantifying the various bacterial contents, it is possible to understand how the gut microbiota and health of newborns are related. Very few studies have examined different selective agar media with a specific focus on quantifying, isolating and growing bifidobacteria in faecal samples [2].

An antibiogram was made after evaluating Man Rogos Sharpe (MRS), Brain Heart Infusion (BHI), Luria Bertani (LB) and Bifidobacteria agar for both the enumeration, isolation and growth of bifidobacteria in infant faecal samples (breast-fed and formula-fed infants). RB (Raffinose *Bifidobacterium *) was developed for the isolation and enumeration of bifidobacteria from poultry and caecal samples [3, 4], and different media, such as Beerens agar [5–7], were recommended for obtaining bifidobacteria from the gut microbiota. This further indicates that different media are needed for the isolation of bifidobacteria from different sources. Mupirocin is only present in just MW agar, which is species specific and insensitive to several lactobacilli but resistant to bifidobacteria [8]. All the media stated and used are complex and cost-effective. Hence, it is essential to study the growth and isolation of *Bifidobacterium* spp. in a simpler, commonly used universal medium. One of the primary objectives of the present study was to understand, optimize, isolate and identify *Bifidobacterium* spp. in the common media and, subsequently, to develop an antibiogram.

According to bifidobacteria population density studies (of the gut), Yildrim *et al*. [9] reported that 25 % of the adult and 95 % of the new-born/infant gut microbiota (primarily) consists of *Bifidobacterium* spp. The members of the various species of *B. catenulatum, B. bifidum, B. infantis, B. brevi,* and *B. adolescentis *, etc., along with other probiotic microbes, are used as probiotic supplements in the form of sachets, tablets and in different foods, especially in beverages. Through the spread of resistance genes such as plasmids and insertion sequence elements, the widespread use of antibiotics in recent decades has caused the establishment of antibiotic resistance in bacterial species as well as the emergence of antibiotic-resistant diseases [10–12]. Bifidobacteria having antibiotic resistance may horizontally transfer the responsible antibiotic resistance gene to harmful and pathogenic microbes, which may further lead to the formation of not only antibiotic-resistant pathogens, but also result in the failure of antibiotic treatment. Based on these relevant incidents and reports, it is critical to investigate and develop a bifidobacterial antibiogram to differentiate between resistant and susceptible varieties, which will also aid in understanding the type of resistance, such as intrinsic or acquired. Antibiotic-associated diarrhoea is the most common adverse reaction to antibiotic use. It is well known that antibiotic therapy not only reduces the viability of indigenous bifidobacteria [13] but also significantly affects the intestinal microbial flora. Previous reports have concluded that administering *B. longum* reduced antibiotic-related diarrhoea and resulted in a reduction in the time required for recovery from rotavirus diarrhoea [14]. A recent reports by Dhanashree *et al*. [15] stated that bifidobacteria can also be used for antibiotic and probiotic therapy, specifically for tuberculosis infection. Therefore, the successful prophylactic use of bifidobacteria must be able to do more than only alleviate gastrointestinal conditions in order to be effective in treating intestinal diseases, compete with, suppress and eliminate pathogens, but also circumvent and tolerate antibiotic treatment.

The viability of bifidobacteria may be affected by intestinal environmental factors such as low pH, the presence of bile salts and alkaline conditions. During their transit through the gut, they alter both their physiological functions and susceptibilities to antimicrobials. Before considering them for prophylactic applications, a thorough investigation of their antibiotic susceptibilities and resistance before and after gastrointestinal passage is required. In the present study, we propose the development of an antibiogram in three different test media, in particular because there are no suitable and standard protocols available for the susceptibility testing of bifidobacteria. In addition, we propose to examine changes in susceptibilities to multiple antimicrobial agents between different isolates due to stress caused by alkaline, acidic conditions and hydrogen peroxide (H_2_O_2_).

In this study, bifidobacterial isolates and strains, mostly of human and dairy origin, were tested against antibiotics of different functional classes. Eleven different types of antibiotics were used: amoxyclave, chloramphenicol, kanamycin, pristinomycin, vancomycin, β-lactams, macrolides, tetracycline and amino glycosides. With the help of the standard and particular lactic acid bacteria (LAB), these strains were all tested by using E-test techniques. To test the antibiotic susceptibility of non-enterococcal LAB, susceptibility test medium [16] (LSM) [Lipase Salt Mannitol] [16]) was used. The study focused on a number of bifidobacteria strains with resistant and susceptible phenotypes distinguished by the amplification of antibiotic resistance genes.

## Methods

Between December inghood hospitals. Inclusion requirements were: age under 6 months at study entry was a requirement for inclusion; male or female; symptoms of cow milk allergies;and no regular probiotic supplements taken (for more than 1 week and within 6 weeks before entering the study). In total, 284 (66 %) of the 431 newborns recommended by neighbourhood health clinics had parents who were interested in taking part. In total, 230 infants (ages 3–6 months, mean 6.4) out of the 252 who met our inclusion criteria were included. Each infant’s parent gave their assent, individually. In this investigation, parents of term infants (*n*=371) were contacted. All newborns began to nurse, and we included those who began formula feeding within 4 weeks after birth. Those who changed to formula-feeding within 4 weeks after birth were randomly assigned to one of the two formula groups. Growth, stool characteristics and side effects that occurred in recruited infants were recorded in a 3 month follow-up period. Nurses in the hospital collected faecal samples from a sub-population and mothers in the homes of infants for analysis of intestinal bacteria (culture technique), acetic acid (GC) and pH (indicator strip). Of the 100 samples, only ten were further screened. The ten faeces samples were subcultured on non-selective and selected medium, respectively, from breastfeeding and formula-fed infants at 4, 30 and 90 days postpartum. For each sample, freshly voided faecal material from different infants was/were collected and pooled. Table 1 provides a list of all the different bacteria that were isolated and used in the investigation. Paediatric departments provided the vast bulk of the faeces samples that were collected (hospitals). Of these, three samples represent (F1–F3 in Table 1) four different paediatric departments, and the remaining samples (F4–F6 in Table 1) represent formula-fed infants in and around the area.

**Table 1.** Bacterial counts of total culturable anaerobic bacteria (A), bacterial counts obtained for the selective agar media (B), and number of bifidobacteria isolates (B), recovered from: F1 to F3, breast-fed infant faecal samples; F4 to F6, formula-fed infant faecal samples

Sample	Bacterial counts of total culturable bacteria (c.f.u. g^–1^)	LB agar			BHI agar			MRS agar			Bifido agar		
		A	B	C	A	B	C	A	B	C	A	B	C
F1	8×10^9^	3.5×10^9^	25	1	9×10^9^	25	8	3×10^5^	25	1	8.5×10^9^	22	10
F2	7×10^8^	4×10^8^	22	0	6×10^8^	25	2	2×10^7^	22	0	9×10^7^	22	9
F3	9×10^9^	8×10^7^	25	1	7×10^9^	23	5	3×10^7^	23	0	9×10^9^	25	8
F4	6×10^7^	3×10^5^	25	0	3×10^8^	25	1	2×10^5^	25	0	4×10^9^	25	0
F5	5×10^7^	3×10 ^7^	24	0	5×10^9^	23	0	2×10^6^	22	0	5×10^8^	25	1
F6	6×10^9^	3×10 ^4^	24	0	7×10^9^	23	0	2×10^5^	20	0	8×10^9^	22	0

Total culturable anerobic bacteria.

A: Bacterial counts, B: No. of isolates investigated, C: No. of Bifidobacteria identified.

CFU, Colony forming units.

The processing of 5–10 g of faeces was done in an aerobic environment under sterile conditions. With the use of anaerobic Wilkins-Chalgreen agar, the total number of anaerobic bacterial cultures was counted (Difco 1805-17-6). The cultures were inoculated and maintained in an anaerobic gas jar for 5 days at 37 °C. Serial dilutions on BHI, LB, MRS and Bifidobacteria agar were then used to count and isolate bifidobacteria. Twenty-five colonies from the highest dilution of each sample were randomly selected from each selective agar. They were then inoculated with hemin at a concentration of 0.005 g l^–1^ and placed in reinforced Clostridium broth (Merck 5411). The isolates were kept as glycerol stocks at −70 °C. Isolate subcultures were grown at 37 °C in 10 ml trypticase-peptone-yeast extract broth. A genomic DNA isolation kit (Macherey-Nagel) was used to extract the genomic DNA after centrifuging growing cells at 9000 ***g*** for 10 min as instructed by the manufacturer.

### Identification of the novel isolate *B. catenulatum*

Table 2 contains a list of the numerous bifidobacteria strains and isolates used in this investigation. All the listed strains were confirmed as bifidobacteria with a species-specific PCR targeting a partial and complete 16S rDNA sequence (650 and 1500 bp) using the technique outlined by Alander *et al*. [17] before being subjected to antibiotic susceptibility testing [17]. The bifidobacteria isolates were grown at 37 °C in an atmosphere-creating anaerobic gas jar. Before testing for antibiotic sensitivity, cultures were cultivated on LSM plates overnight to allow them to become acclimated to the test media. Isolated and individual colonies were then re-suspended in fresh 2.0 ml of sterile 0.9% saline until an OD_600nm_ of 0.16–0.20 (equivalent to McFarland standard 1.0) was attained.

**Table 2.** *
Bifidobacterium* strains used in this study

Bacterial strain	Strain no.	Reference
* B. animalis* subsp. *lactis *	DSMZ 10140	[31]
* B. thermacidophilum* subsp. *thermacidophilum *	DSMZ 15837	[32]
* B. adolescentis *	DSMZ 20083	[33]
* B. longum* subsp. *infantis *	DSMZ 2008	[33]
*B. asteroids* 1969	DSMZ 20089	[34]
* B. animalis *	DSMZ 20105	[35]
* B. breve *	DSMZ 20213	[33]
* B. indicum *	DSMZ 20214	[34]
* B. catenulatum *	Faecal isolate from a 14-month-old baby	This study
*B. atenulatum*	Faecal isolate from a 14-month-old baby	This study

### Growth of bifidobacteria under aerobic and anaerobic conditions

Bifidobacteria are obligate anaerobes; they are sensitive to oxygen and cannot grow under aerobic conditions. Therefore, they require anaerobic conditions for growth, essentially in their initial phase of growth. Similar conditions to those for anaerobic cultivation were set and followed for the cultivation of bifidobacteria under aerobic conditions as well. However, small changes were introduced: first the initial growth phase was set at low pH, second, and more importantly, the partial removal of oxygen from the system before and after autoclaving, and finally by the introduction of 1.0 % of N_2_ gas into the media.

### Antibiotic susceptibility determination

Following the E-test protocol, a sterile cotton swab was first dipped in the inoculum (about 3×10^8^ c.f.u. ml^−1^) and manually dispersed on the MRS/Bifido/TPY/MH agar plates. The plates were then allowed to dry for 15–30 min before the E-test strip was applied (Hi-Media Laboratories). The MIC values for ten different antibiotics, including inhibitors of protein synthesis (gentamycin, GEN10; tetracycline, TET30; chloramphenicol, CHL30; erythromycin, ERY15), inhibitors of cell wall synthesis (amoxicillin/amoxyclave, AMX10), and other widely used antibiotics such as ampicillin, tetracycline, kanamycin, pristomycin, streptomycin and vancomycin (obtained from Invitrogen), were determined for ten different bifidobacteria (Table 1).

The E-test (biodisk) method was followed to develop an antibiogram on different media such as Brucella agar, LSM agar supplemented with cysteine, TPY and MH agar. Susceptibility or resistance testing were performed according to the NCCLS [18]. The concentrations employed in the E-test were 0.016–256 g ml^−1^ for all antibiotics. Finally, the E-test agar plates underwent a 48 h anaerobic incubation in a gas jar at 37 °C (Hi-Media Laboratories; portable anaerobic workstation). Following incubation, the MICs were calculated and determined in triplicate for each strain.

### Influence of Oxgall or bile salt, hydrogen peroxide and low pH tolerance on MIC

The method of Gagnon *et al*. was followed [19]. In brief, sterile 96-well ELISA plates (BD-biosciences) were seeded with various bifidobacteria. Each strain was treated with bile salts, hydrogen peroxide and low pH. The results are presented as the MIC of bile salts, hydrogen peroxide and pH that completely inhibit the growth of the organism.

### Effect of bile salts, hydrogen peroxide and low pH challenge on antibiogram

An antibiogram was developed for isolates that were subjected to different concentrations of bile salts (0.3 % physiological concentration), hydrogen peroxide (1.0–2.0 %) and low pH (pH 3.0). MICs for these bacteria were determined on MRS media only, considered as a standard medium based on the above studies. Mid-log phase cultures were harvested from 5–10 ml of cultures at 4.0 °C, and the pellet was then re-suspended in equal amounts of fresh MRS broth that had been pH-adjusted to 3.0 with concentrated HCl. The remaining pellet was re-suspended in H_2_O_2_ at its MIC, while a second fraction of the isolates were re-suspended in MRS broth with 0.3 % (w/v) bile salts and pH 6.5. The cells were incubated anaerobically for 60 min at 37 °C and for 90 min with bile salts and H_2_O_2_ for acid challenge studies. All of the aforementioned samples were then diluted in peptone water (0.1 %, w/v) and plated onto MRS agar before being incubated at 37 °C for 12–16 h to create an antibiogram.

### Fructose 6-phosphate hosphoketolase (F6PPK) assay

The method of Orban [20] was followed. In brief, the isolated pure *Bifidobacterium* cultures were grown in MRS broth (Hi-Media) to stationary phase at 37 °C and under anaerobic conditions. Subsequently, cells were harvested by centrifugation at 10000 r.p.m. and 4.0 °C for 10–12 min (Remi centrifuge) and treated twice with phosphate buffer to remove any debris. They were then extensively rinsed with CTAB (180 g) for effective lysis before being incubated at room temperature for 10–12 min. The second step involved the addition of a solution containing sodium fluoride (NaF), fructose 6-phosphate (F6P) and potassium acetate (KoAc) (750, 20 and 1.25 µg, respectively), and then incubated for 5–10 min. Finally, samples were treated with H_3_NO/HCl (195 mg), and incubated at room temperature for 10 min. Finally, samples were treated with H_3_NO/HCl (195 mg), and then left at room temperature for 10 min. TCA (150 mg), 4.0 M HCl (1.0 ml) and ferric chloride were added to the reaction to precipitate the contents of the reaction as well as the components of cell lysis. Ferric chloride was then added to create colour (50 mg FeCl_2_). The reaction was subsequently incubated for 10 min at room temperature. The appearance of a reddish violet colour indicates the presence of *Bifidobacterium *, and yellow colour indicates absence.

### PCR amplification

PCR amplifications were performed to find the potential genes for ampicillin, erythromycin, streptomycin and tetracycline resistance. Further, the alkaline lysis method for plasmid DNA isolation was followed to understand whether the factors responsible for antibiotic resistance are of plasmid or chromosomal origin. In addition, genes coding for very common antibiotic resistance were identified for chloramphenicol, kanamycin, tetracycline and vancomycin, as they are very common antibiotics and are prescribed by physicians for bacterial infections. The primers and oligonucleotides used to represent the antibiotic resistance genes are listed below.

- Resistant genes of amino glycoside: *aac[5]-1a, aac[5]-1b, aac[5]11a*.
- Resistance genes of β-lactam: *bla (ACC-01-03*).
- Resistance genes of chloramphenicol: *cat 1, cat 2, cat 3,* etc.
- Resistance genes of macrolides linosamide, streptogramin: *ere (a), ere (B), mef (A), msr (B), sat (A), vat (A*).
- Resistance genes of sulfonamide: *sul1*, *sul2.*
- Resistance genes of tetracycline: *tet(21), tet(31*).
- Trimethoprim resistance genes: *drfA, A1.*
- Vancomycin resistance genes: *vanA, vanB.*

To understand the origin of genes responsible for antibiotic resistance such as of intrinsic or plasmid origin, these were followed by PCR amplification with standard primers and PCR. As a template, purified genomic DNA from several bifidobacteria was employed. PCR was followed with the corresponding primers for different antibiotics by using *pfu* DNA polymerase. The following parameters were used: denaturation at 95 °C for 45 s, renaturation at 52 °C for 30 s, extension at 72 °C for 60 s and final extension at 72 °C for 4.0 min. Sequencing of the amplified material was done after it had been analysed using agarose gel electrophoresis.

### Isolation of plasmid DNA

Only for isolates that exhibited antibiotic resistance was plasmid DNA isolated using the conventional technique. Briefly, overnight-grown bacteria were extracted, lysed using a lysozyme solution, and then solution I; 25 mM Tris/HCl (pH 8.0), 10 mM EDTA, solution II; 200 mM NaOH, 1% SDS, solution III; 3 M Potassium Acetate, pH 5.5 were sequentially added. They were centrufuged for 15–20 min at ambient temperature. The resultant lysate was then exposed to a spin column, effectively washed, and then further eluted using TE buffer (Tris, EDTA). An agar gel electrophoresis was used to determine whether plasmid DNA was present in the resultant elute.

### SEM studies of *B. adolescentis* grown in various media

To understand the effect of various media on the cell morphology of *B. adolescentis *, a single isolated colony was streaked on various media plates such as LB, MRS, BHI and Bifidobacteria agar, followed by incubation for 12–16 h at 37 °C. The isolated single colonies were grown in fresh media and were harvested. The pellet was washed twice with sterile phosphate buffer, fixed with glutaraldehyde and the samples were stored at 4.0 °C. It was then viewed by scanning electron microscopy (SEM; 10× and 20× magnification). The surface patterns of the bacteria grown on various media were also observed by eye.

## Results and discussion

16S rRNA gene sequence analysis confirmed that the novel isolate represents *B. catenulatum *. All the bacterial isolates and type cultures used in the study are detailed in Table 1. The colony forming units/bacterial counts attained in the current study are displayed in Table 2. Counts from the LB, MRS, Bifido and BHI agar media were lower than the total number of cultivable bacteria. In one sample, no colonies were discovered above the detection limit of 1.9×10^5^ c.f.u. g^−1^, and the lowest counts were on MRS agar (Table 1). We also examined the various sizes and morphologies of the colonies on each agar medium.

**Fig. 1.**
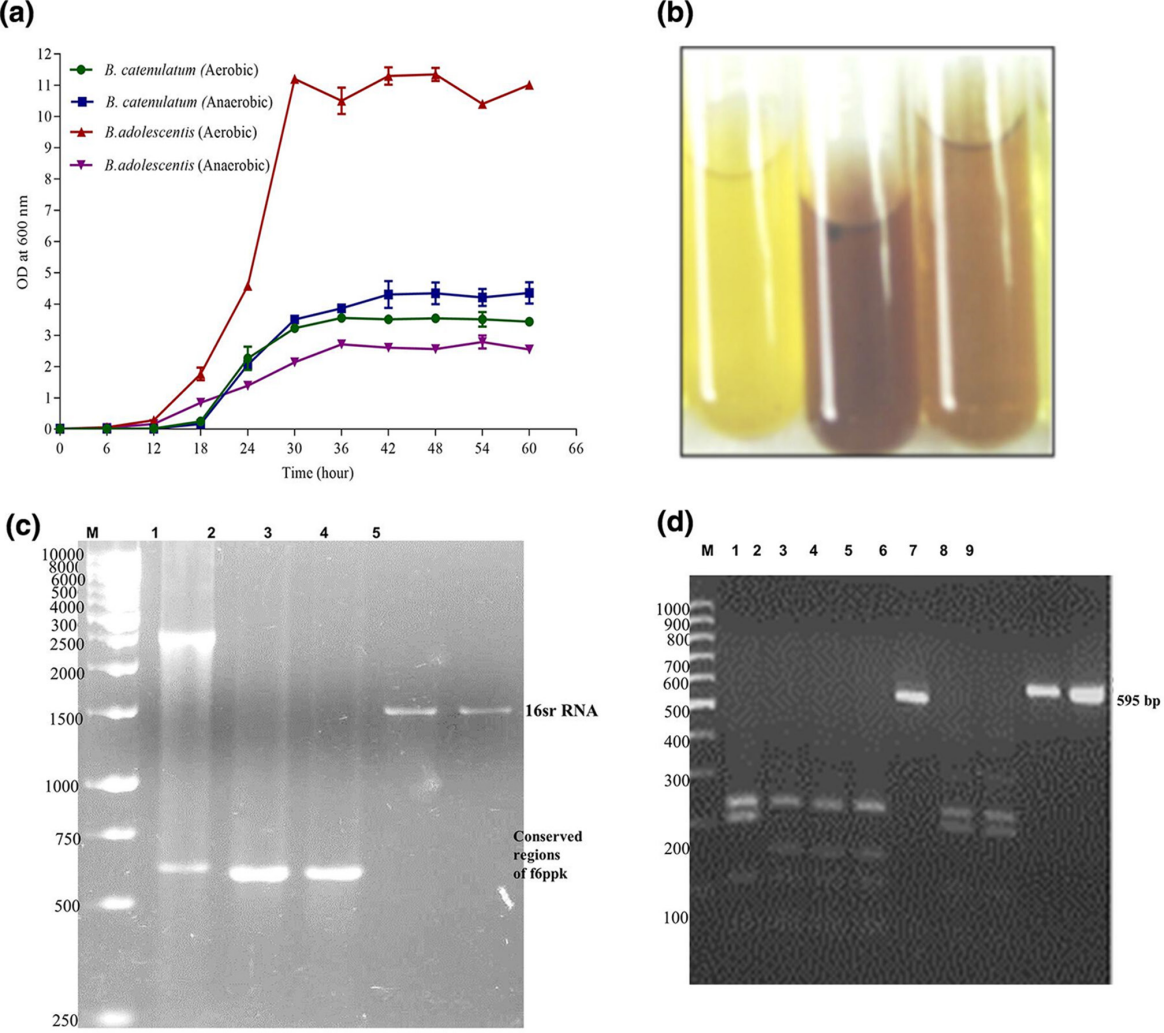
(a) Growth curve of bifidobacteria under aerobic and anaerobic conditions. (b) The Orban *et al*. method for the phosphoketolase assay was used. Collected cells were treated with CTAB after being washed in phosphate buffer, and re-suspended in 1.0 ml of phosphate buffer. To the lysed cells 250 µl each of sodium fluoride and fructose 6-phosphate were added, mixed and vortexed. They were then treated with 1.5 ml hydroxylamine hydrochloride for 30 min at 37 °C. Finally, they were treated with TCA and ferric chloride, and formation of colour was recorded qualitatively. (c) Genomic DNA was purified from respective bifidobacteria, subjected to 16S rRNA amplification (conserved regions as well as whole gene). The same DNA was further used for amplification of the *xfp* gene. The PCR amplified products were separated on 2.0 % agarose gel along with a marker. Lane 1: molecular weight markers of 500, 750, 1000, 2000, 2500, 3000, 4000, 5000, 6000, 8000 and 10000 bp. Lane 2: *xfp* gene product. Lanes 3 and 4: 16S rRNA conserved amplified regions. Lanes 5 and 6: amplified 16S rRNA. (d) RFLP pattern of DNA fragments corresponding to conserved regions of *xfp*. Amplified conserved regions such as the 590 bp PCR product of different bifidobacterial species were single band purified from agarose gel, and subjected to *Xho1* restriction enzyme digestion as directed by the manufacturer. The reaction mixture was separated on agarose gel. Lane M: 100 bp marker. Lane 1: *B. adolescentis* conserved region digested with *Xho1* restriction enzyme. Lane 2: *B. longum longum *. Lane 3: *B. longum infantis *. Lane 4: *B. breve *. Lane 5:. 590 bp product as a control. Lanes 6 and 7. *B*. sp. isolates; Lane 8: PCR product control incubated without enzyme. Lane 9: amplified PCR product.

Growth curve studies were conducted for *B. catenulatum* (the novel isolate) under aerobic and anaerobic conditions. The culmination of the growth curve took around 66 h. The study began with an initial OD of 0.01, as the doubling time is around 24–30 h; therefore, growth over the first 40 h was very slow (there may have been a lag time). After 40 h growth increased, when the log period of growth started, which remained for 20 h. The growth curve threshold at 60 h marked the start of the lag phase, and this period was prolonged and the samples were then isolated. There was a very slight variation in the growth of *B. catenulatum* under aerobic and anaerobic conditions. The log phase under anaerobic conditions was steep, compared with aerobically growing cultures. Overall, the growth rates were slightly different, especially at the log phase stage under aerobic and anaerobic conditions (Fig. 1a). In all these studies, *B. adolescentis* was considered as a control. This further confirms that bifidobacteria may grow under microaerophilic conditions with minor changes such as by removing the existing oxygen in the media, and injection of external N_2_. This does not involve skilled methods, or high-cost equipment/infrastructure facilities such as anaerobic gas chambers.

It was discovered that the ‘bifid’ shunt, which is used to test *Bifidobacterium* spp., is a phosphoketolase reaction in which d-fructose 6-phosphate is metabolized to erythrose 4-phosphate and acetyl-1-phosphate. Hence, the same cultures, and the positive and negative controls were subjected to the standard (F6PPK) assay. The intensity of reddish violet colour formed by different *Bifidobacterium* strains was quantified spectrophotometrically (absorbance at 505_nm_). As a negative control, test tubes without cells and with cells and all other reagents except F6PPK were employed (Fig. 1b, the yellow colour of the control and the reddish brown colour of the test indicating the separated cultures). The results further confirmed that they are bifidobacterial isolates.

The *Bifidobacterium* isolates and the type strains were subjected to PCR amplification of *xfp* using genus-specific primers (Fig. 1c) (Table S1), full-length *xfp*, conserved regions of *xfp* (590 bp) (Fig. 1c, Lanes 1, 2 and 3; and Fig. 1d, Lanes 5, 8 and 9), and full-length 16S rRNA (Fig. 1c Lanes 4 and 5). As expected, the amplifications were observed with an expected DNA. The amplification of various specific and conserved regions of 16S rRNA, and F6PPK with different primers gave an indication that the isolates are bifidobacteria. F6PPK is the marker gene/enzyme found in bifidobacteria species. The presence of the gene further confirms that they are bifidobacteria. Therefore, biochemical and genetic confirmation that the isolates are bifidobacteria were made based on the F6PPK assay and PCR amplification of the relevant gene, respectively.

Additionally, RFLP was applied to the amplified conserved domains of F6PPK. In Fig. 1(d) the RFLP pattern variations are depicted in Lanes 1, 2, 4, 6,and 7. Lanes 1–4 and 6–7 in Fig. 1(d) are different. This suggests that they show several bifidobacteria species. Particularly with the MRS and LB agar, a low recovery of bifidobacteria from the selective agar medium was seen, demonstrating a bias in the counts towards excessively high bacterial levels. According to data from 65 investigations, bifidobacteria are typically found in people in concentrations of (log) 9.0–10.5 per gram of wet weight. In the current study, we looked at the ability of various media to quantify total anaerobes, bifidobacteria, and lactobacilli. Media were chosen based on their selectivity (from data in the literature) and utilization in many investigations. Fresh human faeces from breast-fed and formula-fed infants were used for all testing media. Despite a detection limit of about 4.0 c.f.u. g^–1^, bifidobacteria were not discovered in the faeces of newborns who were fed formula; nevertheless, bifidobacteria were discovered (2.5×10^9^ c.f.u. g^−1^) in the majority of faecal samples from babies who were breastfed.

Similar findings were reported with our samples, and no bifidobacteria were found in a thorough analysis of intestinal bacterial populations based on a library of 401 bacterial 16S rRNA gene sequences cloned from the gut content of 20–25 formula-fed infants. These findings support the findings of the current investigation, which found that the population of bifidobacteria in formula-fed infant gastrointestinal tracts is quantitatively low (Table 1).

In the current investigation, the bifidobacterial isolates were split into two groups based on distinct results from RFLP analysis of the 16S rRNA and *xfp* gene (Fig. 1c). The remaining samples were more closely related to *B. adolescentis* compared with Group 1 isolates. *B. longum, B. longum longum*, and *B. brevi* were preserved in the second group based on the RFLP pattern and alignment of the 16S rRNA gene sequence. *B. infantis* and *B. longum* are predominantly of human origin, according to previous research. This is the first instance where all isolated strains of *B. catenulatum* have been recognized as being of human origin.

Almost all common sugars, with the exception of glucose amine, can be fermented by the isolated new *B. catenulatum *, which was first discovered in breast-fed neonates (Table 3) . They all ferment lactose, galactose, l-arabinose, sodium gluconate, salicin and α-methyl-d-glucoside (Table 3). Mikkelsen *et al*. [21, 22] concluded that neither the type strains nor the bifidobacteria isolated from pigs have the capacity to ferment the aforementioned carbohydrates. Consequently, we view the *Bifidobacterium* spp. isolated from breast-fed newborns as being exceptional in several ways. The ability of these isolates to digest lactose may be linked to the newborns’ increased dietary intake of lactose from breast milk.

**Table 3.** Fermentation patterns of bifidobacteria isolated in this study and the type strain of *B. breve
*

Substrate	Bifidobacterial isolates		* B. breve* (DSM)
	Group I	Group II	
Lactose	+	+	+
Xylose	+	+	+
Maltose	+	+	+
Fructose	+	+	+
Dextrose	+	+	+
Galactose	+	+	+
Raffinose	+	+	+
Trehalose	+	+	+
Melibiose	+	+	+
Sucrose	+	+	+
l-Arabinose	+	+	+
Mannose	+	+	+
Inulin	+	+	+
Sodium gluconate	+	+	+
Glycerol	+	+	+
Salicin	+	+	+
Glucosamine	–	–	–
Dulcitol	+	+	+
Inositol	+	+	+
Sorbitol	+	+	+
Mannitol	+	+	+
Adonitol	+	+	+
α-Methyl-d-mannoside	+	+	+
Xylitol	+	+	+
ONPG	–	–	–
Aesculin hydrolysis	–	–	–
d-Arabinose	+	+	+
Sorbose	+	+	+
Citrate utilization	–	–	–
Malonate utilization	–	–	–

The present study’s low selectivity of bifidobacteria on MRS and LB agar showed that these media are insufficient for counting bifidobacteria in breast-fed infant faeces. On LB and MRS agar, bifidobacteria are seen as separate, tiny, milky colonies, although lactobacilli have comparable traits. Therefore, when isolating from LB and MRS agar in the current investigation, we found it challenging to recognize these traits, and these criteria were not taken into account. The extraordinarily low quantity of bifidobacteria seen in both media may be partially explained by this phenomenon. The only media that permitted recovery were those containing bifidobacteria and BHI, which displayed the highest quantity of bifidobacteria (Table 3) . Breast-fed and formula-fed infant faeces were used to identify non-bifidobacterial isolates, and Table 4 lists their distribution on LB, BHI, MRS and Bifidobacteria agar. These findings might help in the development of improved selective agar for counting bifidobacteria in the future. Table 5 displays the antibiogram of bifidobacteria in various media. The remaining antibiotic MIC values fell within a constrained range (Table 5).

**Table 4.** MICs of antibiotics for bifidobacteria

Organism†	MIC (µg ml^–1^) of the following antibiotics* for the indicated organism on MH media									
	Amo	Amp	Chl	Ery	Gen	Kan	Pri	Str	Tet	Van
* B. adolescentis *	0.5	0.016	0.1	0.25	0.01	3.0	1.0	1.0	0.01	0.5
* B. animalis *	>240.0	0.512	0.1	0.1	0.25	3.0	1.0	5.0	0.1	0.05
* B. asteroides *	>240.0	0.512	0.1	0.1	0.25	3.0	0.1	5.0	0.1	0.1
* B. breve *	>240.0	0.512	0.1	0.5	0.25	5.0	1.0	3.0	2.0	0.1
* B. indicum *	0.2	0.032	0.1	0.5	0.1	1.0	1.0	1.0	0.01	0.05
* B. infantis *	1.0	0.032	0.01	0.5	0.1	1.0	1.0	1.0	0.01	0.1
* B. longum *	>240.0	8.0	1.0	0.25	0.5	3.0	1.0	3.0	1.0	0.1
*B. thermoacidophilum*	>240.0	0.512	0.1	0.5	0.25	5.0	1.0	7.5	1.0	0.1
Isolate 1	>240.0	0.512	0.5	0.25	0.25	3.0	1.0	3.0	1.0	0.1
Isolate 2	>240.0	0.256	0.5	1.0	0.25	3.0	1.0	3.0	0.5	0.1

*Amo, amoxycillin; Amp, ampicillin; Chl, chloramphenicol; Ery, erythromycin; Gen, gentamicin; Kan. kanamycin; Pri, pristomycin; Str, streptomycin; Tet, tetracycline; Van, vancomycin.

†*Bifidobacterium*.

**Table 5.** MIC values (µg ml^–1^) of *Bifidobacterium* spp. on different media

Antibiotic	Brucella agar			TPY agar			Bifido agar		
	* B. longum* *	* B. catenulatum* †	* B. catenulatum* †	* B. longum* *	* B. catenulatum* †	* B. catenulatum* †	* B. longum* *	* B. catenulatum* †	* B. catenulatum* †
Amoxyclave/amoxicillin	>240	>240	>240	>240	>240	>240	>240	>240	>240
Ampicillin	8	16	32	4	16	8	8	32	32
Chloramphenicol	1	2	1	0.5	1	0.5	1	1	1
Erythromycin	0.1	0.1	0.1	0.1	0.1	0.1	0.5	0.25	0.25
Gentamicin	0.25	0.25	0.25	2	5	5	1	1	0.5
Kanamycin	3	1	3	10	30	15	7.5	7.5	3.0
Pristomycin	1	0.1	1	0.1	0.1	0.1	1	1	1
Streptomycin	3	1	3	30	30	30	15	10	10
Tetracycline	1	0.25	0.1	1	1	0.25	1	1	1
Vancomycin	0.1	0.5	0.5	0.5	0.5	0.5	0.5	0.5	0.5

*Control strain.

†Novel isolate from faecal material of a 7-month-old infant.

Notably, *Actinomyces* spp. were found in the isolate from a baby who was fed formula. Because these also grew exceptionally well on LB and displayed the distinguishing bifid-shaped cellular morphology, the use of morphology for identifying bifidobacteria on the designated agar medium was insufficient. According to the data shown in Table 6, the isolates stand out in biochemical tests such as nitrate reductase and Voges–Proskaeuer. Definitive assessment of the presence of bifidobacteria on the selective agar media generally followed the F6PPK assay and *xfp* gene amplification. As previously confirmed by genus-specific *in situ* hybridization [21] and DGGE, the 16S rRNA-specific primers and genus-specific primers have extremely precise target areas within the 16S rRNA gene of bifidobacteria [23].

**Table 6.** Biochemical test patterns of bifidobacteria isolated in this study and the type strain of *B. breve
*

Name of the test	Bifidobacterial isolates		* B. breve* (DSM)
	Group I	Group II	
1. ONPG	–	–	–
2. Lysine utilization	–	–	–
3. Ornithine utilization	–	–	–
4. Urease	–	–	–
5. Phenylalanine deamination	–	–	–
6. Nitrate reductase	+	–	–
7. HS production	–	–	–
8. Citrate utilization	–	–	–
9. Voges–Proskaeuer	–	+	–
10. Methyl red	+	+	+
11. Indole	–	–	–
12. Malonate utilization	–	–	–

The current study has shown that, when applied to infant faeces samples, LB and MRS agar media are insufficiently selective for bifidobacteria, but Bifidobacteria agar and BHI agar indicate superiority with respect to both selectivity and sensitivity. The findings also show that there is a minimal population of bifidobacteria in the faeces of newborns who have been fed formula. The isolated *Bifidobacterium* strains from breast-fed neonates are dominated by *B. catenulatum *. A potential new *Bifidobacterium* species was also discovered, which we hope to characterize in more detail in our upcoming research.

**Fig. 2.**
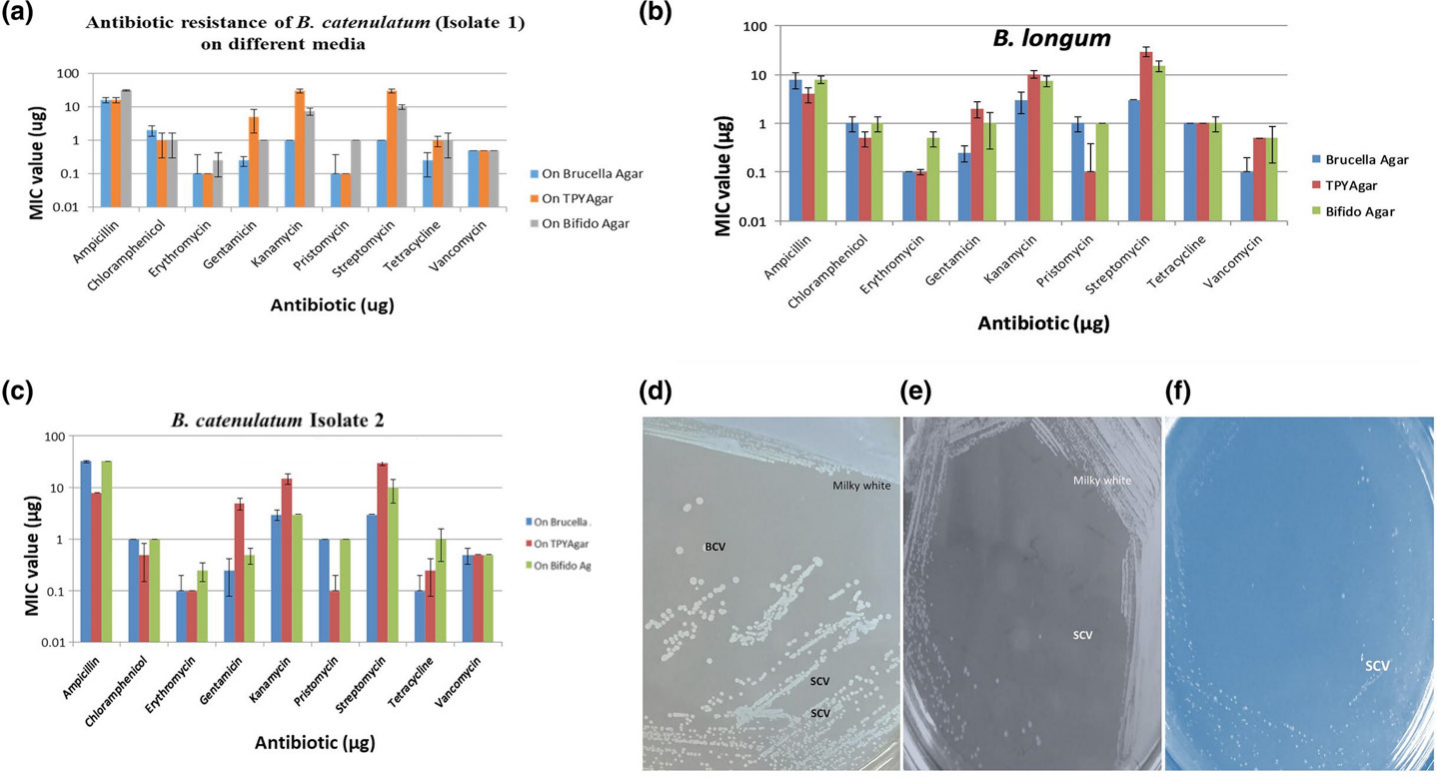
(a), Antibiotic resistance of *B. catenulatum* (Isolate 1) on Brucella agar, TPY and Bifido agar. (b) Antibiotic resistance of *B. longum* on Brucella agar, TPY and Bifido agar. (c) Antibiotic resistance of *B. catenulatum* (Isolate 2) on Brucella agar, TPY and Bifido agar. (d) Naked eye observation of bifidobacteria grown in MRS medium. (e) Naked eye observation of bifidobacteria grown in BHI medium. (f) Naked eye observation of bifidobacteria grown in LB medium.

All the strains of *Bifidobacterium* showed good growth on Bifidobacterial, and MH (Mueller Hinton Hi-Veg media, Hi-Media laboratories) agar and most strains on TPY under anaerobic as well as aerobic conditions (Fig. 1a growth curve under aerobic and anaerobic conditions). For BRU (Brucella agar) susceptibility testing, certain bifidobacterial strains did not grow well. Some of them on BRU displayed little growth. All of the strains in the study were determined to be *Bifidobacterium* spp. based on results from species-specific PCR amplifications. In the presence of antibiotics such as ampicillin, kanamycin and erythromycin, these bacteria displayed pinpoint colonies occasionally observed with the E-test method (Fig. 2d–f). During the process, we observed the formation of pinpoint bifidobacteria after treatment with amoxyclave. These pinpoint colonies were observed irrespective of the media used for antibiogram studies. There is a possibility that the formation of pinpoint colonies indicates their susceptibility to the antibiotic exposed. This means there is a close relationship between the formation of microcolonies and antibiotic exposure. In these circumstances, the MICs were successfully established a fourth time. A control strain obtained from the DSM culture collection centre along with a few further multi-drug-resistant strains were tested and we successfully developed an antibiogram. The formation of pinpoint colonies and the above phenomenon may indicate pseudo-resistance, meaning no single element is responsible for the resistance. Tthe presence of a tough cell wall may restrict diffusion of the antibiotic.

**Fig. 3.**
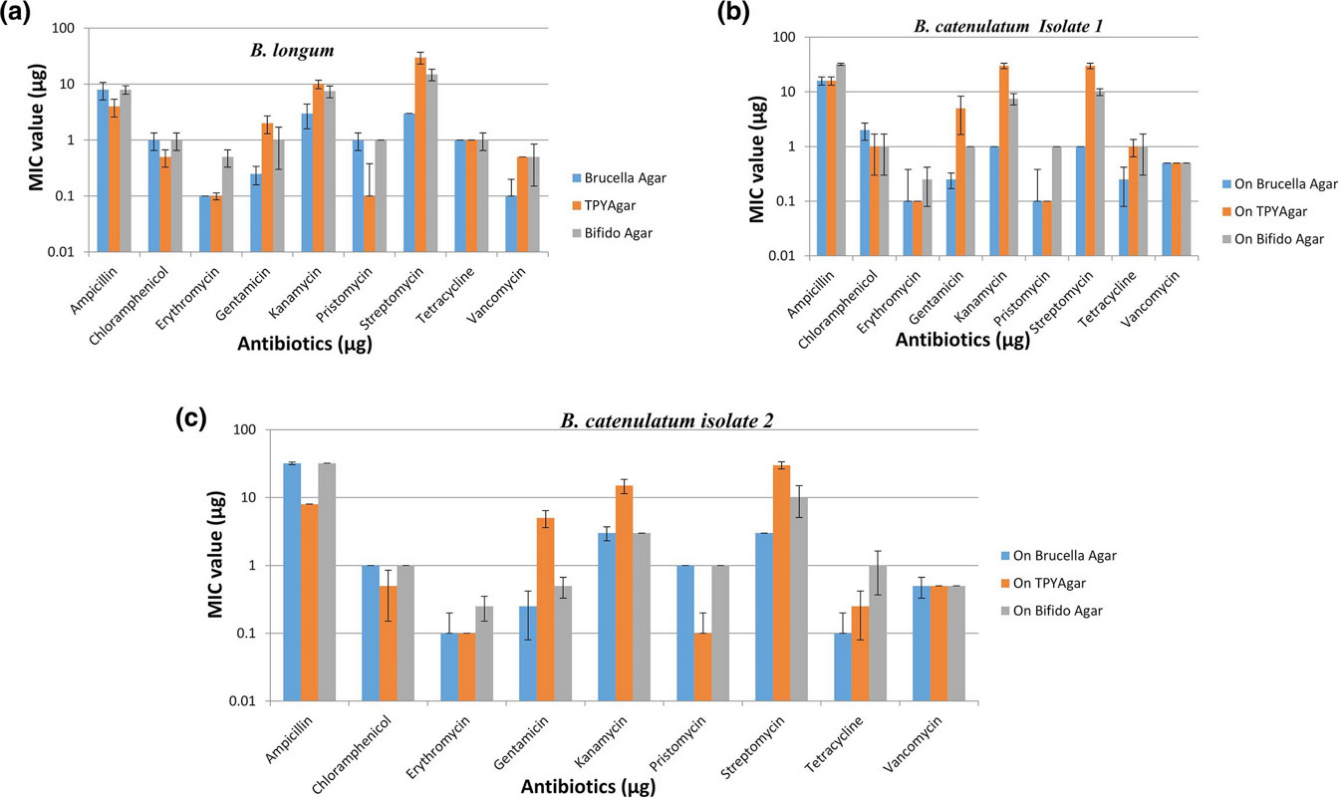
(a) MIC values of . *B. longum* grown on Brucella agar, TPY agar and Bifido agar in the presence of various antibiotics. (b) MIC values of *B. catenulatum* (Isolate 1) grown on Brucella agar, TPY agar and Bifido agar in the presence of various antibiotics. (c) MIC values of *B. catenulatum* (Isolate 2) grown on Brucella agar, TPY agar and Bifido agar in the presence of various antibiotics.

All of the bifidobacteria strains tested positive for amoxyclave resistance, two strains tested positive for ampicillin resistance and two strains tested positive for streptomycin resistance, as assessed by an E-test alone, in accordance with previous findings of antibiotic resistance thresholds and the microbiological breakpoint criteria established by the panel on additives and products or chemicals used in animal feed (FEEDAP) (2008) [24]. Examples of E-test results on different supplemental agar media are shown in Fig. 3(a–c) and Table 4. The results were compared and analysed with the previous bifidobacteria antibiogram reports [25, 26].

**Fig. 4.**
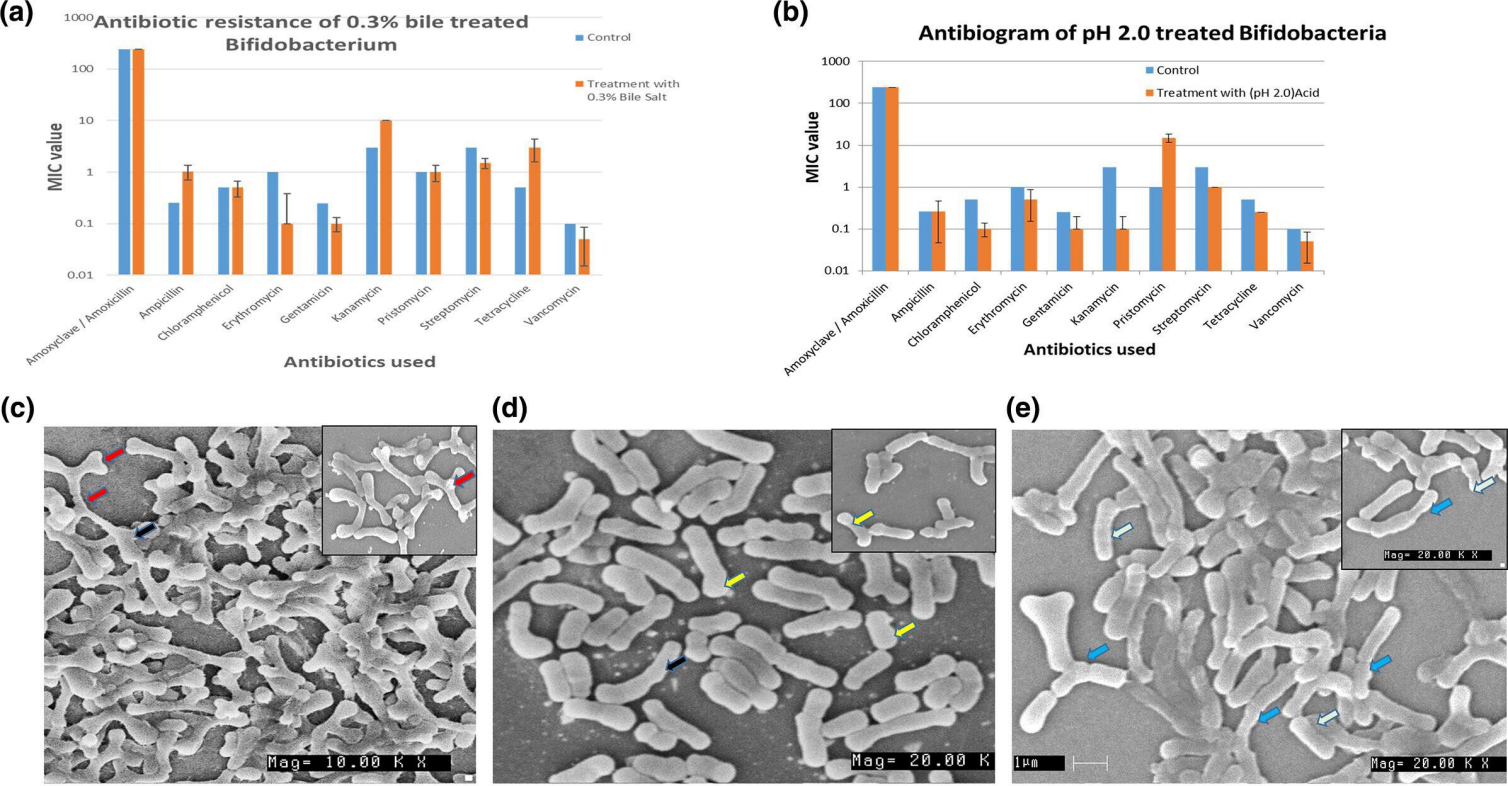
(a) Antibiotic resistance of 0.3% bile-treated *B. catenulatum.* (b) Antibiogram of pH 2.0-treated *B. catenulatum* (Isolate 1). (c) *B. adolescentis* grown in MRS media, Inset, 20× magnification. Red arrow indicates bifid shape. Dark coloured arrows indicate big colony variants (BCV). (d) *B. adolescentis* grown in LB media, Inset, 20× magnification showing isolated colonies. Yellow arrows indicate rod shape. (e) *B. adolescentis* grown in BHI media, Inset, 20× magnification showing isolated colonies. Blue arrows indicate un-divided abnormally long bifidobacteria. Light green arrow indicates small colony variants (SCV).

The results of the PCR amplification were inconsistent with the phenotypic patterns of the studied species. Amoxycillin, ampicillin and streptomycin resistance genes are known to occur, but PCR was unable to identify them. Despite being phenotypically resistant to these antibiotics, the type-bacteria behaved similarly to the test strains. Some bacteria displayed peculiar traits by displaying ampicillin resistance on Bifido and BRU media, but the same strains were shown to be sensitive on TPY media (Fig. 3a–c). Similarly, a few strains showed sensitivity to kanamycin on Bifidobacteria media but were resistant on TPY (Tables 3 and 4). Similar findings with streptomycin were found with the three different media. The results pertaining to MIC values for different antibiotics such as amoxyclave, chloramphenicol, erythromycin, gentamycin, pristomycin, tetracycline and vancomycin on different media (Bifido, TPY and BRU) were identical (Table 4, Fig. 3a–c). MIC values of ten different bifidobacteria strains on MH agar were not only consistent but also in the range previously reported (Table 4). The MIC values for amoxyclave, ampicillin and vancomycin showed good correlation between the test media. The MIC values for ten different bifidobacteria strains on MH agar were consistent, except those for amoxyclave (Table 4, Fig. 4a, b). Bifidobacteria were treated with 0.3 % bile and subjected to pH 2.0, and subsequently exposed to various antibiotics to understand their resistance before and after treatment. We observes that the MIC values did not change with regard to amoxicillin, chloramphenicol, streptomycin and pristinomycin. The values were almost identical before and after treatment. MIC values for bifidobacteria exposed to pH 2.0 did not change with regard to ampicillin or amoxicillin, but slight variations were observed where MIC levels decreased after exposure to low pH and bile when the cell membrane is compromised and therefore the intake will be rapid and the MIC should decrease. MIC values of *Bifidobacterium* spp. on different media are shown in Table 5. The same phenomenon was observed in most. If they have developed intrinsic resistance the phenomenon may be reversible (Fig. 4a, b).

Bifidobacteria grown on various media such as MRS, LB and BHI were subjected to SEM which revealed that only MRS-grown bifidobacteria were bifid in shape (Fig. 4c–e). The bacteria grown in LB media were rod shaped, and no bifid shapes were observed (Fig. 4c–e). Similarly, the bacteria grown in BHI were elongated rod shaped, and no bifid shapes were observed (Fig. 4c–e). This further confirms that MRS is a suitable medium for growth as it does not influence cellular morphology. This is in accordance with previous reports of the influence of media on bacterial cellular morphology.

MIC values in different media containing different antibiotics differ from strain to strain, as well as medium to medium. MIC values of MH media are much lower than with the other media. The MIC values for *B. longum* and our isolates in Bifido agar, BRU, TPY and MH agar were different. MIC values were higher in Bifido agar in comparison with the other media (Fig. 4a–c, Table 4). The MIC values were much lower in MH agar in comparison with the other media. The values differed very drastically for ampicillin, streptomycin and kanamycin (Fig. 2a–c) . The remaining MIC values were within a constrained range (Table 5) and MIC values for three different strains with MH media are in the reported range. *B. adolescentis* is one of the most sensitive microorganisms showing sensitivity to all the antibiotics (Fig. 4a, b, Table 4), but it is intrinsically resistant to anti-tubercular drugs [27].

To understand the antibiotic resistance of bifidobacteria exposed to 0.3 % bile and pH 2.0, Figs 2(a, b) and 4(a–c)) demonstrate that samples treated with 0.3% bile had much lower MIC levels but continued to exhibit antibiotic resistance. The MIC values or resistance go down when subjected to low pH. This phenomenon would be anticipated with a compromised cell wall at low pH. Therefore, they will be susceptible/sensitive to the antibiotics (Figs 2 and 4). The phenomenon with bile-treated samples is unexplained, as no reports exist on how bile salts are involved in compromising the cell wall.

### Cell morphological analysis

Colonies appeared (with the naked eye) on MRS agar to be very small (resembling the tip of a pin), delayed in growth and milky white. However, colonies on BHI agar plates were large and milky white (Fig. 2d) . These observations confirm that the media components influence bacterial colony size and texture. The colonies on LB agar were much smaller, and no large colonies were observed (Fig. 2d–f). Bifidobacteria grown in different media show different growth patterns; SCV and BCV types were observed only on MRS. A very slow growth pattern was observed on LB.

## Conclusion

It is challenging to distinguish between closely related species of the genus *Bifidobacterium *, with the exception of *B. brevi*’s capacity to thrive under aerobic conditions, such as at pH 6.0 of the medium and with the addition of 0.2% glucose in oxygen-depleted and N_2_-containing media. As a result, the majority of the bifidobacteria thrived in the newly created aerobic environment (Fig. 1). To identify a species or strain, modern biology techniques such as species-specific PCR-DGGE, TGGE or PFGE are useful tools [28, 29]. The validity and authenticity of each of the strains employed in this investigation, listed in Table 2, has been established. Trans-aldolase has been used in previous investigations [30] to identify, find, and enumerate bifidobacteria [30]. We used infant faecal samples for the present study. Most oexisting procedures for the isolation, identification and enumeration of bifidobacteria depend on sophisticated, laborious and costly methodologies. We focus on very simple and cheaper technologies through which one can isolate, identify and enumerate bifidobacteria spp from various sources.

All of the media, including Bifido, MH, TPY and MRS agar, allowed the bifidobacteria to proliferate. Additionally, it was simple to understand the MIC endpoints that were acquired using the E-test on MH agar. Last but not least, this study shows that the MIC values achieved by using various media can vary. This leads to the further conclusion that test settings also affect the microbiological breakpoints that distinguish between resistant and susceptible organisms. Some of the PCR amplification results and the absence of plasmid DNA were in conflict with the phenotypic patterns of the studied species. Simple PCR amplification and plasmid presence did not reveal known ampicillin, amoxyclave or streptomycin resistance factors. Controls showed the expected outcomes even though the bacteria were phenotypically resistant to these medicines. Three phenotypically sensitive strains showed a hybridization signal with an oligonucleotide that represents *bla* (ACC-01), *bla* (ACC-02) and *bla*, according to further southern hybridization studies and their analysis (ACC-03). One of the isolates we used in this analysis also appeared to have the streptomycin resistance-coding phosphotransferase gene (*aphE*). Since there were no available control strains for these particular genes, validation of these data was unsuccessful; as a result, it is likely that these findings are false positives. We were puzzled to see various sizes of bacteria when they grew on MRS agar. The colonies were very small and milky white. However, they were large and milky white on BHI agar. A previous report [28] clearly envisaged that SCV phenotypes are the survival strategies of bacteria during stress conditions, here theantibiotic exposure [27]. To date, this phenomenon has been reported only in intracellular pathogens such as *Salmonella* Typhi, *Mycobacterium tuberculosis* and *Listeria monocytogenes* . The identical phenomenon as observed in bifidobacteria*,* which is non-pathogenic, and a probiotic. 16S rRNA comparison of SCVs and the wild-type established that there is no variance. Therefore, it is essential to develop tools to differentiate them. Very many fewer and small isolated colonies were observed on LB agar, and no BCVs were observed (Fig. 2d–f).

## Supplementary Data

Supplementary material 1
