# Supplementary material for: Acquiring bifidobacteria species from formula-fed and breast-fed newborns: identifying, quantifying and creating an antibiogram
Source: Access Microbiol. 2023 Aug 8;5(8):acmi000590.v3. doi: 10.1099/acmi.0.000590.v3 (PMC10484311; doi:10.1099/acmi.0.000590.v3)
Supplement: Supplementary material 1 [file acmi-5-590.v3-s001.pdf]

SI No	Name of the primer and Sequence
1	16srRNA forward primer $^{5'}$AGA GTT TGA TCC TGG CTC AG $^{3'}$
2	16srRNA reverse primer $^{5'}$ TAC GGT TAC CTT GTT ACG ACT T$^{-3'}$
3	Specific primer: 16s Bifido forward $^{5'}$ CTC CTG GAA ACG GGT GG $^{3'}$
4	16s Bifido reverse $^{5'}$ GGT GTT CTT CCC GAT ATC TAC A $^{3'}$
5	16s Bifido forward $^{5'}$ GGG TGG TAA TGC CGG ATG $^{3'}$
6	16s Bifido reverse $^{5'}$ CCA CCG TTA CAC CGG GAA $^{3'}$
7	16s Bifido forward $^{5'}$ GGG TGG TAA TGC CGG ATG $^{3'}$
8	16s Bifido reverse $^{5'}$ CCA CCG TTA CAC CGG GAA $^{3'}$
9	16s forward $^{5'}$ CTC CTA CGG GAG GCA GCA G $^{3'}$
10	16s forward $^{5'}$ GTA GCG GTG AAA TGC GTA GA ^{3,}
11	16s forward $^{5'}$ AAA CTC AAA GGA ATT GAC GG-^{3}
12	Reverse 16s $^{5'}$ GGG CGG AGT GTA CAA GGC $^{-3}$
13	Reverse 16s $^{5'}$ GGG TTG CGC TCG TTG $^{-3}$
14	Reverse 16s $^{5'}$ TCT ACG CAT TTC ACC GCT AC-$^{3'}$
15	Bifidobacterium specific primers<br>16s Bifido forward $^{5'}$ CTC CTG GAA ACG GGT GG$^{-3'}$
16	16s Bifido reverse $^{5'}$ GGT GTT CTT CCC GAT ATC TAC A$^{-3'}$
17	16s Bifido forward $^{5'}$ GGG TGG TAA TGC CGG ATG-$^{3'}$
18	16s Bifido reverse $^{5'}$ CCA CCG TTA CAC CGG GAA-$^{3'}$
19	16s Bifido forward $^{5'}$ GGG TGG TAA TGC CGG ATG-$^{3'}$
20	16s Bifido reverse $^{5'}$ CCA CCG TTA CAC CGG GAA-$^{3'}$
21	F6PPK forward $^{5'}$ CCA TGA ACC TGC TCG TCT CCT CCC ACG TGT-$^{3'}$
22	F6PPK reverse $^{5'}$ GAC AAG CCG GTG CTG TTC GCT TAC-$^{3'}$
23	F6PPK reverse $^{5'}$ CGG TGC TTG ACG TCT TCG CGG GTG AAA GGC-$^{3'}$
24	F6PPK forward $^{5'}$ CCA TGA ACC TGC TCG TCT CCT CCC ACG TGT-$^{3'}$
25	F6PPK forward $^{5'}$ GAC AAG CCG GTG CTG TTC GCT TAC$^{-3'}$
26	F6PPK reverse $^{5'}$ CGG TGC TTG ACG TCT TCG CGG GTG AAA GGC$^{-3'}$

Supplementary Table 1.Primers used in this study.
